# Medication Errors in Intensive Care Units: An Umbrella Review of Control Measures

**DOI:** 10.3390/healthcare10071221

**Published:** 2022-06-29

**Authors:** Sara Dionisi, Noemi Giannetta, Gloria Liquori, Aurora De Leo, Victoria D’Inzeo, Giovanni Battista Orsi, Marco Di Muzio, Christian Napoli, Emanuele Di Simone

**Affiliations:** 1 Department of Biomedicine and Prevention, Tor Vergata University of Rome, 00133 Rome, Italy; sara.dionisi@uniroma1.it (S.D.); gloria.liquori@uniroma1.it (G.L.); aurora.deleo@uniroma1.it (A.D.L.); 2 School of Nursing, UniCamillus—Saint Camillus International University of Health and Medical Sciences, 00131 Rome, Italy; noemi.giannetta@uniroma1.it; 3 Nursing, Technical, Rehabilitation, Assistance and Research Direction, IRCCS Istituti Fisioterapici Ospedalieri—IFO, 00144 Rome, Italy; emanuele.disimone@uniroma1.it; 4 Department of Clinical and Molecular Medicine, Sapienza University of Rome, 00185 Rome, Italy; victoria.dinzeo@yahoo.it (V.D.); marco.dimuzio@uniroma1.it (M.D.M.); 5 Department of Public Health and Infectious Diseases, Sapienza University of Rome, 00185 Roma, Italy; giovanni.orsi@uniroma1.it; 6 Department of Surgical and Medical Sciences and Translational Medicine, Sapienza University of Rome, 00185 Rome, Italy

**Keywords:** medication errors, prevention, patient safety, intensive care units

## Abstract

Medication errors are defined as “any preventable event that may cause or lead to inappropriate medication use or patient harm while the medication is in the control of the health care professional, patient, or consumer.” Such errors account for 30 to 50 percent of all errors in health care. The literature is replete with systematic reviews of medication errors, with a considerable number of studies focusing on systems and strategies to prevent errors in intensive care units, where these errors occur more frequently; however, to date, there appears to be no study that encapsulates and analyzes the various strategies. The aim of this study is to identify the main strategies and interventions for preventing medication errors in intensive care units through an umbrella review. The search was conducted on the following databases: PubMed, CINAHL, PsycInfo, Embase, and Scopus; it was completed in November 2020. Seven systematic reviews were included in this review, with a total of 47 studies selected. All reviews aimed to evaluate the effectiveness of a single intervention or a combination of interventions and strategies to prevent and reduce medication errors. Analysis of the results that emerged identified two macro-areas for the prevention of medication errors: systems and processes. In addition, the findings highlight the importance of adopting an integrated system of interventions in order to protect the system from harm and contain the negative consequences of errors.

## 1. Introduction

There is no univocal definition of medication errors (MEs). The most recent definition was given by the National Coordinating Council for Medication Error Reporting and Prevention [1]; it defined MEs as “any preventable event that may cause or lead to inappropriate medication use or patient harm while the medication is in the control of the healthcare professional, patient, or consumer”. The consequences of MEs affect patient safety and may cause patient injury, disability, and death due to failures in healthcare facilities. In addition to the human factor, one of the more systems-focused causes of the increase in MEs is the process of corporatization that has contaminated health systems since the 1990s [2]. The corporatization of health care facilities has led to process management similar to that found in factories; in addition, lawsuits by citizens against health care professionals have increased, leading to an increased aversion to clinical risk and its reporting on the part of health care personnel. In this context, MEs impose a heavy and preventable burden on health care systems, accounting for 30% to 50% of all healthcare errors [3,4]. Thus, healthcare systems have focused their attention on the risk posed by various healthcare personnel in healthcare settings, and they have put in place management and control procedures to prevent errors. 

Moreover, errors are often related to not just one factor but rather a series of events that have the potential to overcome all barriers put in place to avoid harm or discomfort to the patient. The allegorical Swiss Cheese Model proposed by Reason [5] facilitates the analysis and identification of the causal factors of errors. Safety barriers implemented to prevent errors, such as those related to procedures and controls (the “slices of cheese”), regulate and protect the functioning of the system from damage and its consequences, allowing the timely identification of anomalous processes [6]. Several studies and literature reviews try to individuate “slices of cheese”, i.e., systems or interventions that have the potential to reduce the frequency at which MEs occur. Some authors have focused on specific factors, such as excessive workload, fatigue, the proprieties of certain therapies [7], a culture of belonging [8,9], specific healthcare settings [10,11], and healthcare environmental contamination [12,13,14].

Limited research has compared the prevalence or incidence of MEs occurring in different wards, as opposed to research on single wards [15,16,17]. The literature contains a large number of studies focused on the intensive care unit setting (ICU) [18]. Indeed, patients in ICUs receive medications mostly through their veins; this often requires the calculation of infusion drop rates. In addition, these patients are mostly in poor condition or unconscious and unable to monitor and report adverse drug reactions; therefore, the prevalence and consequences of MEs increase in this setting [19]. Moreover, some contributing factors are related to operators, such as nurses, and their working conditions [20], organizational climate, occupational characteristics, the physical aspects of the work environment (poor lighting, poor thermal and acoustic aspects), and a high number of patients [21]. Therefore, in considering these factors, it is useful to describe and identify all the available strategies to improve the safety of patients [22]. Despite the large body of evidence and multiple reviews available on this topic, to our knowledge, no umbrella review has been conducted to summarize them, especially for the ICU setting.

Thus, this umbrella review aims to identify strategies and interventions to prevent MEs in ICUs. The research question is as follows: what interventions prevent MEs in ICUs?

## 2. Materials and Methods

This umbrella review was conducted based on guidelines elaborated by the Joanna Briggs Institute (JBI) [23,24]. The registration number of this umbrella review protocol on the PROSPERO registry is CRD42021235767. The PRISMA checklist is presented in Supplementary file S1.

### 2.1. Literature Search

A preliminary search was conducted using the following electronic databases: Medline (Pubmed), Cumulative Index to Nursing and Allied Health (CINAHL), Embase, Scopus, and PsycInfo.

The literature search also included an analysis of the bibliographic references used to trace systematic literature reviews referenced by authors that could be relevant for the current review. To be useful to decision-makers, reviews should aim to be as comprehensive as possible. Therefore, the following keywords were used: ME, intervention(s), strategies, systematic review, and meta-analysis. Regarding the definition of ME, the authors utilized the one offered by the National Coordinating Council for Medication Error Reporting and Prevention [1] and reported in the Introduction. The search strategy for each database is presented in Supplementary file S2.

In order to investigate the main interventions in the literature, no time limits were set. Specifically, all studies published up to 31 May 2022 were considered eligible. In addition, a language limit was imposed, considering only studies published in Italian and English. The following criteria of inclusion and exclusion were set:

- Participants: all systematic reviews of literature that had target populations of healthcare professionals involved in the prescription, distribution, or administration of medication in adult ICUs were included. These professionals included nurses, pharmacists, and physicians of any medical discipline or specialty. Systematic reviews that concerned nursing students and/or trainees of any healthcare discipline were excluded.
- Interventions: all systematic reviews that evaluated the efficacy of interventions aimed at preventing or reducing MEs were included.
- Outcomes: all systematic reviews that reported MEs and evaluated error rates, incidence, or prevalence as their primary or secondary outcome were included.
- Setting: all systematic reviews that analyzed interventions or strategies put in place in the ICU were included. Authors defined an ICU as all of the units that cater to the healthcare needs of patients in critical condition who require a high level of intensity of care.
- Study typology: only systematic reviews and/or meta-analysis studies were included.

### 2.2. Study Selection

All the references that were collected from our search on various databases were exported from said databases and imported into the Mendeley Reference Manager^®^ software (Mendaley Desktop 1.19.8; London, UK) package. Then, all duplicates were removed.

Consequently, each title and abstract were evaluated based on the inclusion and exclusion criteria fixed a priori; this was carried out autonomously and independently by two reviewers. All doubts or controversies were resolved through the comparison and intervention of a third reviewer.

Thereafter, the full texts of the reviews that satisfied the inclusion criteria were acquired and then subjected to the first two reviewers. The two reviewers screened the full-text articles retrieved and then independently assessed the eligibility of each one. In case of a disagreement between these two authors, a third reviewer resolved the disagreement. Some full-text articles were removed, stating the reason for their exclusion; others were included in the umbrella review. Any further disagreement was resolved by a discussion between all three reviewers. If the full texts were not available online or in libraries, the authors of the reviews were contacted.

### 2.3. Assessment of Quality

For each systematic review included, the quality of the methodology was assessed autonomously and independently by two reviewers using the JBI Critical Appraisal Checklist for Systematic Reviews and Research Syntheses [23,24]. Disagreements were resolved by a third reviewer.

### 2.4. Data Extraction and Synthesis

For each included systematic review, the following information was extracted: authors, title, year, type of studies included, number of studies included, number of participants included, description of the intervention, description of the outcomes, research strategy, instrument used for the evaluation of methodological quality, and main results. The results were aggregated according to emerging categories and are presented in the form of a table (Table 1). Information not present in the included studies is reported in Table 1 as “Unreported”.

Extracted findings are presented in tabular format for each intervention. In addition, each primary study included in each systematic review was tabulated to assess the overlap between reviews (Table 2, Table 3, Table 4, Table 5 and Table 6). The advantages and disadvantages of each intervention were investigated, tabulating the findings of each systematic review into categories labeled positive, negative, or not statistically significant (Table 7).

## 3. Results

### 3.1. Study Selection

**Figure 1:**
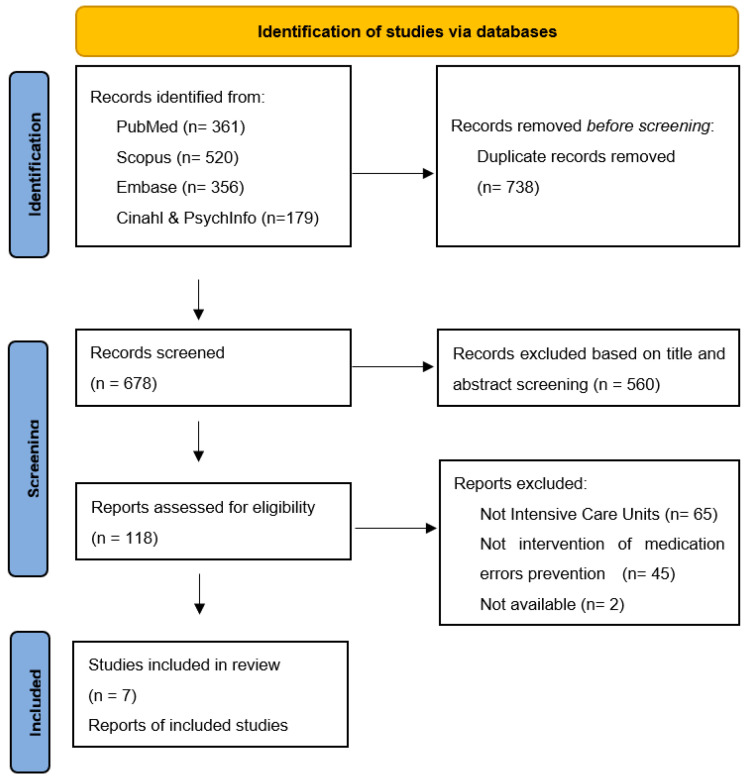
Flow diagram of the search and selection process, based on the PRISMA flowchart. From [32].

In the preliminary phase of the research, 1416 citations were identified (Figure 1). Of these, 738 were duplicates and subsequently removed. Of the remaining 678 citations, two reviewers independently screened the literature by reading the titles and the abstracts, consequently removing 560 citations.

Only 116 of the 118 potentially eligible full texts were available. Three of the authors of the unavailable full texts were contacted without receiving an answer. Conclusively, seven of the systematic revisions were considered eligible.

Figure 1 shows the search and selection process according to the PRISMA statement [32].

### 3.2. Characteristics of the Studies Included

Seven systematic reviews were included (five systematic reviews and two systematic reviews with meta-analysis) for a total of 47 studies. All reviews aimed to evaluate the efficacy of a single intervention or a combination of interventions and strategies for the prevention of and reduction in MEs. The majority of the reviews included did not report the number of participants recruited either in the main text or in the data extraction tables. Overall, 25 studies included were conducted in the United States of America; 4 studies were conducted in the United Kingdom; 2 studies each were conducted in Australia, Belgium, Canada, Germany, and the Netherlands; and others were conducted in China, Brazil, Egypt, France, Malaysia, Spain, Switzerland, and Vietnam. With regard to study design, 38 primary studies had prospective designs, and most of them were pre-post interventional studies; the remaining 9 studies had retrospective designs.

The analysis of the procured articles allowed for the individuation of two macro-areas of intervention: systems and processes to prevent MEs. Table 1 presents the extractions from the studies included. Specifically:

- by “systems”, we refer to technologies: computerized physician order entry (CPOE), smart infusion pumps, team members, e.g., pharmacists, and organizational factors, e.g., staff working shifts.
- by “processes”, we refer to medication review and medicine reconciliation.

### 3.3. Technologies to Prevent MEs

The majority of systematic reviews included showed that there are a lot of technologies to prevent medication errors at every stage of the pharmaceutical process.

CPOE is defined as “an application that electronically accepts medical prescriptions, substituting the traditional prescription registered manually in the clinical documentation” [33] and was identified as an intervention to reduce MEs in the prescribing stage in four systematic revisions, for a total of 10 included studies [25,26,27,28]. Table 2 shows the studies included in each systematic review.

With a search strategy focused on studies conducted prior to 2011, Manias, Williams, and Liew [26] identified five studies that analyzed the efficacy of CPOE in reducing MEs. The results were heterogeneous: three studies demonstrated an actual reduction in ME rates after implementing CPOE [34,35,36], whilst the remaining two studies reported a rise in MEs, probably due to the introduction of predefined options that can be bypassed by the prescriber or by the numerous other personnel; such defaults are sometimes ignored by medical personnel [37,38]. Similar results concerning an intensive care unit were found in a systematic review conducted by Reckmann, Westbrook, Koh, Lo, and Day [27].

The effectiveness of the use of CPOE in terms of prescribing error rates was documented by a systematic review and meta-analysis by Prgomet, Li, Niazkhani, Georgiou, and Westbrook [25], in which most of the included articles reported an 85% reduction in error rates, albeit with moderate significance (pooled RR: 0.15, 95% CI: 0.03–0.80, *p* = 0.03). However, it must be specified that the extreme heterogeneity of the studies is due to different definitions of error and, above all, the fact that different methods were used to evaluate the error rate. There were significant differences in the prevalence of errors in the different studies, varying from 4.5% to 58.2% in the pre-CPOE phase and from 0% to 8.2% in the post-CPOE phase. Similar results were also reported by van Rosse et al. [28], who highlighted the possible beneficial impacts of implementing CPOE; such an implementation also consists of training healthcare personnel. 

By “systems of support of clinical decisions”, we refer to all technologies and/or strategies that facilitate the decision-making process of healthcare staff (e.g., a computer-assisted antibiotic-dose monitor and clinical information support) and/or technologies that facilitate the distribution process or administration of medication (e.g., automatized systems for distribution and barcode technologies). A systematic review analyzed these instruments for preventing and reducing the incidence of medication errors [26]. These included:

- Barcode technology. This technology allows the electronic identification of patients and the cross-checking of medication details, patient data, the hour of administration, and the staff that executed the administration, based on checking the four Rs: right dosage, right drug, right time, and right administration of the drug. In their systematic review, Manias et al. [26] included a study that showed the real efficacy of this technology for reducing medication errors in the administration and dispensing stages [39].
- The use of automatized systems for the distribution of medication was examined in one study [40]. Based on its findings, the implementation of an automatized system for the distribution of medication did not have beneficial and/or protective effects concerning MEs during the dispensing stage.
- Technological systems for endovenous infusions (smart pumps). The efficacy of these systems for reducing error rates was not documented in the two studies included by Manias et al. [26]. The two studies showed an error reduction rate that was not statistically significant (4.78 vs. 4.95 per 1000 patients per day, *p* = 0.96; 2.03 vs. 2.41 errors per 100 patients per day, *p* = 0.124) [29,40].
- Support systems for clinical decisions, such as the computer-assisted antibiotic-dose monitor. Results from the five included studies demonstrated a statistically significant reduction in medication errors after the implementation of this new technology.

Table 3 lists the studies included for each intervention in the systematic review considered.

### 3.4. Processes to Prevent Medication Errors 

Two systematic reviews [26,29], for a total of 11 included studies, analyzed the efficacy of medicine reconciliation for reducing MEs (Table 5).

The systematic review conducted by Manias et al. [26] included only one relevant study and therefore does not allow us to establish its actual efficacy [41]. The process of medicine reconciliation was addressed by Rice et al. [29], who examined processes undertaken exclusively by pharmacists or pharmacy technicians and not other healthcare professionals. The results of the studies included in their review allow the assertion that the presence of a pharmacist during medicine reconciliation in the transportation from an intensive care unit to another unit is statistically relevant; it resulted in a higher recognition of MEs, as well as a supply of recommendations for bettering treatment plans for the patient. Conversely, the results from the studies that required the intervention of a pharmacist only in the presence of medication or specific pathologies were not relevant.

### 3.5. Team Members to Prevent MEs

Aside from the crucial role pharmacists play during the medicine reconciliation process, two systematic reviews [26,30], for a total of seven articles, analyzed the role of pharmacists during daily clinical care activities regarding the reduction in error risk rates. Manias et al. [26] include four studies that explored the involvement of pharmacists during daily activities (e.g., during review meetings, consults, and rounds). The results appear to be in conflict: two studies showed an effective reduction in the incidence of errors when a pharmacist was present on wards, one study showed an increment in the error rate, and another study had an uncertain result. For clarification, in the systematic review and meta-analysis conducted by Wang et al. [30], the intervention of a pharmacist during daily activity was not statistically associated with a reduction in ME rates (pooled OR: 0.61, 95% CI: 0.11–3.55, *p* = 0.33).

Three systematic reviews [26,30,31], with a total of eight studies, analyzed different educational methodologies to increase pharmaceutical knowledge and, consequently, a reduction in medication errors. Table 4 lists the studies included in the considered systematic reviews. The review conducted by Manias et al. [26] considered two studies. One documented the efficacy of simulation in the reduction in the ME rate with statistically relevant differences between the pre- and the post-simulation results [42]. The other documented the efficacy of field training for the containment of medication prescription errors [43]. The review conducted by Hunter et al. [31] paid particular attention to the preparation and administration of vasoactive medication, frequently used in intensive care areas. Wang et al. [30], on the other hand, evaluated the potential of educational sessions provided directly by pharmacists.

### 3.6. Organizational Interventions to Prevent Medication Errors

A systematic review [26] explored the impact of organizational factors on the incidence of MEs. One of the factors considered was that of staff working shifts. According to the results of the systematic review conducted by Manias et al. [26], an improved organization of work shifts could be a positive and protective model in regard to error rates. The study included in the said systematic review demonstrated a significant reduction in error rates when comparing professionals who worked following a rotation of shifts composed of prolonged work shifts (99.7 errors per 1.000 patients per day) and those who worked a reduced total number of hours per week (82.5 errors per 1.000 patients per day).

However, further research is necessary to confirm these data [44]. The systematic review furthermore measured the efficacy of the use of guidelines and posters for the standardization of clinical care practice and reductions in ME rates. These results are statistically significant in each of the three studies included by Manias et al. [26]. The list of studies included in this systematic review is presented in Table 6.

### 3.7. Quality Assessment

For each systematic review included, the methodological quality was measured using the JBI Critical Appraisal Checklist for Systematic Reviews and Research Syntheses. The minimum number of criteria met was 6, and the maximum was 9 out of 11. The results are listed in Table 7. No reviews were excluded based on methodological quality criteria. Specifically, six out of seven systematic reviews included were of high quality, and only one was of medium quality [23,24]. However, only two systematic reviews stated that the likelihood of publication bias had been assessed (criterion 9). 

## 4. Discussion

This umbrella review aims to identify strategies for preventing MEs that have documented efficacy in ICUs. To this end, the main electronic scientific databases were consulted, procuring systematic reviews from the literature.

The analysis of the results of the included systematic reviews allowed for the identification of interventions that demonstrated effectiveness in reducing MEs. Specifically, a classification of macro-areas of interventions was stated (systems or processes). 

The majority of the systematic literature reviews included in this umbrella review looked into the advantages and disadvantages of the use of a CPOE system, also taking into account computerized solutions to prevent prescription errors. The actual efficacy of the use of CPOE in terms of a reduction in prescription error rates was documented by the systematic review and meta-analysis conducted by Prgomet, Li, Niazkhani, Georgiou, and Westbrook [25], in which the majority of articles included reported a statistically significant reduction in the percentage of errors. However, a review of the primary studies reveals a significant heterogeneity in both the definition of ME used by researchers and the methods used to estimate error rates. Indeed, as affirmed by Prgomet et al. [25], some studies indicated that missing weight information or the lack of a signature constituted an error of omission, and some others included rule violations, while other studies did not list these elements in their error definitions. Moreover, the studies included in this systematic review showed significant differences in the percentage of errors pre- and post-CPOE-introduction.

Apart from these considerations, a significant overall reduction in medication prescribing error rates was demonstrated after the implementation of CPOE in ICUs. This finding is not surprising; indeed, it is well known that the automation and standardization of the prescribing of medication reduce ME rates. 

CPOE and smart infusion pumps could reduce the rate of medication errors in ICUs, but their implementation should be supported by organizational strategies and educational training. Indeed, while technology can reduce error rates, the greatest challenge in all healthcare settings is enabling their appropriate and correct use [25].

Toward this aim, examinations of the pharmacist’s role and integration into the team were widely documented in the included reviews. Indeed, the pharmacist may play a crucial role in increasing knowledge about medicines and patients and consequentially in reducing the ME rate at the prescription or administering stages. To this date, however, there is very scarce evidence regarding the presence and appropriate role of pharmacists in the ward (e.g., whether it should be a permanent presence and include making rounds with the physician or on-call staff). 

Moreover, due to the complexity of clinical settings and the sources of MEs, we must recommend not only one intervention to prevent medication errors but rather an integrated system that includes several safety barriers that allow for the prompt identification of anomalous processes; such barriers should intervene to protect the system from possible damage and contain the negative consequences of anomalies. To that end, future research should be conducted to study changes in error rates resulting from the implementation of both technologies and processes. A unique convergence of empirical observations, statistical findings, and theoretical reflections is found in a perspective that emphasizes the sharing of information in international programs, identifying the most effective strategies, and optimizing the therapeutic process [45].

Vigilance on the part of nurses and the adoption of precautionary measures regarding medication errors in ICUs are key factors for preventing medication errors. All selected strategies yield positive effects in clinical practice: the insurance of a safe environment for medication preparation by placing labels; the reduction in distractions and interruptions during medication administration; the implementation of “five rights”; and the mandatory double-checking of medication, e.g., by two separate nurses.

## 5. Conclusions

The analysis of the results of the systematic reviews included allowed for the identification of interventions that reduce medication errors. The findings show a significant overall reduction in medication error rates in ICUs after the implementation of new technology (such as a CPOE, barcode technology, smart pumps, and so on). Indeed, it is well known that the automation and standardization of medication orders reduce medication error rates [46]. However, it was also found that these technologies should be implemented with support systems for clinical decisions and organizational/educational strategies. In fact, it is not a single intervention that should be recommended to prevent medication errors, but rather an integrated system that includes several safety barriers to enable the early identification of abnormal processes and allows taking action to protect the system from possible damage and contain the negative consequences of anomalies. 

Finally, it may be useful to invest in safety during the training of healthcare professionals [47]: this could help reduce not only errors in the hospital setting but also in the home setting [48].

The umbrella review undertaken here presents some limits. First of all, there is the extreme heterogeneity of the definitions of medication errors present in the different studies included in each systematic review. Secondly, there is the heterogeneity present in the methods utilized when measuring error rates, thus making it difficult to compare and, in turn, return strategies of documented absolute efficacy. 

Another limitation of the results is that it is difficult to differentiate the types of errors related to changes in error rates—for example, prescribing, administering, and dispensing medication errors. Some intervention strategies prompt changes at several stages of the pharmaceutical process. In addition, it would be interesting to study the economic evaluation of each intervention included to prevent MEs. However, most of the systematic reviews did not include an economic evaluation. Further research aimed at evaluating the cost-effectiveness of each strategy may provide further evidence on which to base the implementation of control strategies. 

Furthermore, publication bias may be present: there may be unpublished studies that show insignificant results regarding the effectiveness of a given prevention strategy. In order to attempt to address this possible bias, all reference lists were revised. No articles were included as a result of this strategy.

We relied on the umbrella review as a methodological strategy as it allows the achievement of the targeted results/outcomes. However, we are aware that this choice has its limits because it excludes grey literature; to this limit, we also add another one, namely that we excluded studies published in languages other than Italian or English to simplify access to materials. Future development of this work will certainly be oriented toward the removal of these limits. 

In addition, it would be interesting, in future studies, to analyze the results of clinical trials undertaken and not yet concluded to verify the efficacy of emerging strategies. It would also be interesting to bring forward studies that intend to formulate an unequivocal definition of medication errors and establish how such errors are measured to make error rates in different contexts comparable. 

## Figures and Tables

**Table 1:** Summary of findings.

Author(s) (Year)	Title	Study Design	Number of Studies	Number of Participants	Description of Interventions	Description of Outcomes Included in the Review	Search Details	Appraisal Instrument Used	Results
Prgomet et al. (2017) [25]	Impact of commercial computerized provider order entry (CPOE) and clinical decision support systems (CDSSs) on medication errors, length of stay, and mortality in intensive care units: a systematic review and meta-analysis	Unreported	20	Unreported	CPOE and CDSS	Medication errors, length of stay, mortality	Medline and Embase via Ovid, and CINAHL via EBSCOhost	EPHPP quantitative tool	“The transition from paper-based ordering to commercial CPOE systems in ICUs was associated with an 85% reduction in medication prescribing error rates and a 12% reduction in ICU mortality rates. Overall meta-analyses of LOS and hospital mortality did not demonstrate a significant change.”
Manias et al. (2012) [26]	Interventions to reduce medication errors in adult intensive care: a systematic review	Pre-post interventional studies; prospective randomized trials; quasi-experimental designs	24	25 to 8.901	Any interventions delivered in ICUs for adult patients with the intent of reducing medication errors	Error rates	PubMed, CINAHL (Nursing and Allied Health), EMBASE, Journals@Ovid, International Pharmaceutical Abstract Series via Ovid, Science Direct, Scopus, Web of Science, PsycInfo, Cochrane Database of Systematic Reviews, and the Cochrane Central Register of Controlled Trials	Oxford Centre for Evidence-Based Medicine	“Eight types of interventions were identified: computerized physician order entry (CPOE), changes in work schedules (CWS), intravenous systems (IS), modes of education (ME), medication reconciliation (MR), pharmacist involvement (PI), protocols and guidelines (PG) and support systems for clinical decision making (SSCD). Sixteen out of the twenty-four studies showed reduced medication error rates. Four intervention types demonstrated reduced medication errors post-intervention: CWS, ME, MR and PG. It is not possible to promote any interventions as positive models for reducing medication errors. Insufficient research was undertaken with any particular type of intervention, and there were concerns regarding the level of evidence and quality of research. Most studies involved single arm, before and after designs without a comparative control group.”
Reckmann et al. (2009) [27]	Does Computerized Provider Order Entry Reduce Prescribing Errors for Hospital Inpatients? A Systematic Review	Cross-sectional trial; prospective pre- and poststudy; prospective study	3	Unreported	CPOE and CDSS	Medication prescription errors	Ovid MEDLINE, CINAHL, EMBASE, Journals@Ovid, Inspec via Ovid, International Pharmaceutical Abstract Series via Ovid, Cochrane Database of Systematic Reviews, and the Cochrane Central Register of Controlled Trials	Unreported	“Three studies investigated the impact of CPOE on the incidence of prescribing errors in adult ICU patients. One evaluated CPOE with clinical decision support and two without. Two studies found that CPOE (without clinical decision support) was associated with a significant reduction in the prescribing error rate. In one study, prescribing error rates remained unchanged for intermittent drugs and prescribing errors increased for IV fluids and infusions.”
van Rosse et al. (2009) [28]	The effect of computerized physician order entry on medication prescription errors and clinical outcome in pediatric and intensive care: a systematic review	Retrospective cohort; prospective cohort; controlled cross-sectional trial	4	Unreported	CPOE and CDSS	Medication prescription errors, adverse drug events, and mortality	PubMed, the Cochrane Library, and Embase	STROBE (observational studies) and Jadad Tool (experimental studies)	“Meta-analysis showed a significant decreased risk of medication prescription errors with use of computerized physician order entry. However, there was no significant reduction in adverse drug events or mortality rates.”
Rice et al. (2020) [29]	Pharmacy Personnel’s Involvement in Transitions of Care of Intensive Care Unit Patients: A Systematic Review	Prospective randomized controlled trial; prospective cohort comparison studies; a prospective study with a pre- and post-design; two-period study with a retrospective pre-implementation component; prospective postimplementation component; retrospective investigations	10	Unreported	Pharmacist-led intervention	Medication errors, continuation of inappropriate therapies, and interventions on transfer into or out of the ICU	MEDLINE and Embase	Unreported	“A significant improvement was demonstrated with pharmacy-driven intervention in all 4 studies that evaluated the entire ICU patient population. Interventions specific to certain medication and disease improved medication safety measures but were not always statistically significant. Medication error rates are high in patients transferred into and out of the ICU, and limited data exist to address this concern.”
Wang et al. (2015) [30]	Effect of critical care pharmacist’s intervention on medication errors: A systematic review and meta-analysis of observational studies	Non-randomized controlled studies: controlled before and after	8	Unreported	Pharmacist-led intervention	Medication error rates, adverse drug events	MEDLINE, Embase, and Cochrane	Quality Assessment Tool for Before and After (Pre- and Post-) Studies With No Control Group (NIH)	“Results suggest that pharmacist intervention has no significant contribution to reducing general MEs, although pharmacist intervention may significantly reduce preventable adverse drug events and prescribing errors.”
Hunter et al. (2019) [31]	Nurse management of vasoactive medications in intensive care: A systematic review	Observational studies; pre- and post-intervention studies; survey studies; quasi-experimental studies; longitudinal time series; prospective controlled trials; and interviews incorporating content analysis	13	Unreported	Medication education	Risk of medication errors	CINAHL Complete, Medline Complete, and EMBASE	Critical Appraisal Skills Program (CASP) Appraisal Tool for Qualitative Research was used to assess quality	“These four studies indicated that providing education and standardisation of practices could support nursing practice on the preparation of vasoactive infusions and reduce risk for medication errors”

**Table 2:** Studies on CPOE included in the systematic review included in this umbrella review.

Studies	Prgomet et al. (2017) [25]	Manias et al. (2012) [26]	Reckmann et al. (2009) [27]	van Rosse et al. (2009) [28]
Evans et al. (1998a) *		✓	✓	
Thompson et al. (2004) *				✓
Shulman et al. (2005) *	✓	✓	✓	✓
Bradley et al. (2006) *		✓		
Colpaert et al. (2006) *	✓	✓	✓	✓
Weant et al. (2007) *		✓		✓
Carayon et al. (2009) *	✓			
Ali et al. (2010) *	✓	✓		
Armada et al. (2014) *	✓			

* for the studies indicated, please consider the bibliographical references in the columns.

**Table 3:** Studies on interventions to prevent medication administration errors included in the systematic reviews included in this umbrella review.

Studies included	Prgomet et al. (2017) [25]	Manias et al. (2012) [26]
Barcode technology		
DeYoung et al. (2009) *		✓
Automatized systems for the distribution of medication		
Chapuis et al. (2010) *		✓
Technological systems for endovenous infusions		
Rothschild et al. (2005) *		✓
Nuckols et al. (2008) *		✓
Support systems for clinical decisions		
Evans et al. (1998b) *		✓
Evans et al. (1999) *		✓
Fernández Pérez et al. (2007) *	✓	
Fraenkel et al. (2003) *		✓
Rana et al. (2006) *	✓	

* for the studies indicated, please consider the bibliographical references in the columns.

**Table 4:** Studies on educational interventions to prevent medication errors included in the systematic reviews included.

Studies	Manias et al. (2012) [26]	Wang et al. (2015) [30]	Hunter et al. (2019) [31]
Herout et al. (2004) *			✓
Thomas et al. (2008) *	✓		
Ford et al. (2010) *	✓		
Alagha et al. (2011) *		✓	
Jung et al. (2014) *			✓
Nguyen et al. (2014) *		✓	
Melo et al. (2016) *			✓
Tan et al. (2017) *			✓

* for the studies indicated, please consider the bibliographical references in the columns.

**Table 5:** Studies on interventions to prevent errors during medication reconciliation included in the systematic reviews included.

Studies	Manias et al. (2012) [26]	Rice et al. (2020) [29]	Wang et al. (2015) [30]
Medication reconciliation			
Pronovost et al. (2003) *	✓		
Zeigler et al. (2008) *		✓	
Hatch et al. (2010) *		✓	
Coutsouvelis et al. (2010) *		✓	
Hatch et al. (2011) *		✓	
Pavlov et al. (2014) *		✓	
Heselmans et al. (2015) *		✓	
Wills et al. (2016) *		✓	
Bosma et al. (2018) *		✓	
Wohlt et al. (2007) *		✓	
D’Angelo et al. (2019) *		✓	
Pharmacist in the ward			
Leape et al. (1999) *	✓		✓
Lee et al. (2007) *	✓		✓
Kaushal et al. (2008) *			✓
Klopotowska et al. (2010) *	✓		✓
Langebrake et al. (2010) *	✓		
Alagha et al. (2011) *			✓
Jiang et al. (2012) *			✓

* for the studies indicated, please consider the bibliographical references in the columns.

**Table 6:** Studies on organizational interventions to prevent medication errors included in the analyzed systematic reviews.

Studies	Manias et al. (2012) [26]
Work organization	
Landrigan et al. (2004) *	✓
Protocols and guidelines	
Wasserfallen et al. (2004) *	✓
McMullin et al. (2006) *	✓
Bertsche et al. (2008) *	✓

* for the studies indicated, please consider the bibliographical references in the columns.

**Table 7:** Quality assessment of systematic reviews included.

Authors (year)	Q1	Q2	Q3	Q4	Q5	Q6	Q7	Q8	Q9	Q10	Q11
Prgomet et al. (2017) [25]	Y	Y	Y	N	Y	U	Y	Y	Y	U	Y
Manias et al. (2012) [26]	Y	Y	Y	Y	Y	Y	Y	Y	N	Y	Y
Reckmann et al. (2009) [27]	Y	Y	U	Y	U	Y	U	U	N	Y	Y
van Rosse et al. (2009) [28]	Y	Y	Y	N	Y	U	U	Y	N	Y	Y
Rice et al. (2020) [29]	Y	Y	Y	N	U	U	Y	Y	U	Y	Y
Wang et al. (2015) [30]	Y	Y	Y	N	Y	Y	U	Y	Y	Y	Y
Hunter et al. (2019) [31]	Y	Y	Y	N	Y	Y	Y	Y	U	Y	Y

Y = yes; U = uncertain; N = no. Q1: Is the review question clearly and explicitly stated? Q2: Were the inclusion criteria appropriate for the review question? Q3: Was the search strategy appropriate? Q4: Were the sources and resources used to search for studies adequate? Q5: Were the criteria for appraising studies appropriate? Q6: Was critical appraisal conducted by two or more reviewers independently? Q7: Were there methods to minimize errors in data extraction? Q8: Were the methods used to combine studies appropriate? Q9: Was the likelihood of publication bias assessed? Q10: Were recommendations for policy and/or practice supported by the reported data? Q11: Were the specific directives for new research appropriate?

## Data Availability

Not applicable.

## Supplementary Materials

The following supporting information can be downloaded at: https://www.mdpi.com/article/10.3390/healthcare10071221/s1, Supplementary file S1: Prisma Checklist; Supplementary file S2: Search strategy.
