# Supplementary material for: Medication Errors in Intensive Care Units: An Umbrella Review of Control Measures
Source: Healthcare (Basel). 2022 Jun 29;10(7):1221. doi: 10.3390/healthcare10071221 (PMC9320368; doi:10.3390/healthcare10071221)
Supplement: Supplementary file 1 [file healthcare-10-01221-s001.zip › Supplementary File S2.pdf]

Supplementary File S2 Search strategy

No.	Query EMBASE	Results
**#22**	#20 AND #21	332
**#21**	#13 AND #17	7796
**#20**	#18 OR #19	334603
**#19**	'systematic review':ti,ab,kw	211019
**#18**	'metaanalysis':ti,ab,kw OR 'meta analysis':ti,ab,kw OR 'meta-analysis':ti,ab,kw	213139
**#17**	#14 OR #15 OR #16	5230154
**#16**	'system':ti,ab,kw	2905199
**#15**	'strategy':ti,ab,kw OR 'strategies':ti,ab,kw	1413137
**#14**	'intervention':ti,ab,kw OR 'interventions':ti,ab,kw	1358938
**#13**	#3 OR #5 OR #6 OR #7 OR #8 OR #9 OR #10 OR #11 OR #12	17020
**#12**	'near miss':ti,ab,kw OR 'medication safety':ti,ab,kw	6374
**#11**	'discharged summaries error':ti,ab,kw OR 'discharged summaries errors':ti,ab,kw	1
**#10**	'medication administration error':ti,ab,kw OR 'medication administration errors':ti,ab,kw	443
**#9**	'dispensing error':ti,ab,kw OR 'dispensing errors':ti,ab,kw	545
**#8**	'transcribing error':ti,ab,kw OR 'transcribing errors':ti,ab,kw	38
**#7**	'prescribing errors':ti,ab,kw OR 'prescribing error':ti,ab,kw	1445
**#6**	'preventable adverse drug event':ti,ab,kw OR 'preventable adverse drug events':ti,ab,kw	269
**#5**	'medication incident':ti,ab,kw OR 'medication incidents':ti,ab,kw	281
**#4**	'medication incident'	113
**#3**	#1 OR #2	10095
**#2**	'medication errors':ti,ab,kw	8716
**#1**	'medication error':ti,ab,kw	3122
